# Enabling Rural Telehealth for Older Adults in Underserved Rural Communities: Focus Group Study

**DOI:** 10.2196/35864

**Published:** 2022-11-04

**Authors:** Inga Hunter, Caroline Lockhart, Vasudha Rao, Beth Tootell, Samuel Wong

**Affiliations:** 1 School of Management Massey Business School Massey University Palmerston North New Zealand; 2 Vensa Health Auckland New Zealand

**Keywords:** access, choice, trust, telehealth, rural, barriers, enablers, underserved populations, underserved, equity, elder, older adult, eHealth, telemedicine, barrier, enabler, facilitator, focus group

## Abstract

**Background:**

Telehealth is often suggested to improve access to health care and has had significant publicity worldwide during the COVID-19 pandemic. However, limited studies have examined the telehealth needs of underserved populations such as rural communities.

**Objective:**

This study aims to investigate enablers for telehealth use in underserved rural populations to improve access to health care for rural older adults.

**Methods:**

In total, 7 focus group discussions and 13 individual interviews were held across 4 diverse underserved rural communities. A total of 98 adults aged ≥55 years participated. The participants were asked whether they had used telehealth, how they saw their community’s health service needs evolving, how telehealth might help provide these services, and how they perceived barriers to and enablers of telehealth for older adults in rural communities. Focus group transcripts were thematically analyzed.

**Results:**

The term *telehealth* was not initially understood by many participants and required an explanation. Those who had used telehealth reported positive experiences (time and cost savings) and were likely to use telehealth again. A total of 2 main themes were identified through an equity lens. The first theme was *trust*, with 3 subthemes—trust in the telehealth technology, trust in the user (consumer and health provider), and trust in the health system. Having access to reliable and affordable internet connectivity and digital devices was a key enabler for telehealth use. Most rural areas had intermittent and unreliable internet connectivity. Another key enabler is easy access to user support. Trust in the health system focused on waiting times, lack of and/or delayed communication and coordination, and cost. The second theme was *choice*, with 3 subthemes—health service access, consultation type, and telehealth deployment. Access to health services through telehealth needs to be culturally appropriate and enable access to currently limited or absent services such as mental health and specialist services. Accessing specialist care through telehealth was extremely popular, although some participants preferred to be seen in person. A major enabler for telehealth was telehealth deployment by a fixed community *hub* or on a mobile bus, with support available, particularly when combined with non–health-related services such as internet banking.

**Conclusions:**

Overall, participants were keen on the idea of telehealth. Several barriers and enablers were identified, particularly trust and choice. The term *telehealth* is not well understood. The unreliable and expensive connectivity options available to rural communities have limited telehealth experience to phone or patient portal use for those with connectivity. Having the opportunity to try telehealth, particularly by using video, would increase the understanding and acceptance of telehealth. This study highlights that local rural communities need to be involved in designing telehealth services within their communities.

## Introduction

### Background

Telehealth is defined as “healthcare delivered using digital technology where participants may be separated by time and/or distance” [1]. Telehealth has been available for >50 years [2] but had not been widely adopted in New Zealand before the COVID-19 pandemic [3-5], despite its known benefits [6-8]. The use of telehealth has increased during the pandemic [4]. Although telehealth use is now higher than that before the COVID-19 pandemic, it is mainly by phone, and telehealth has not yet been embedded as a *business as usual* option for access to health care [4,8-10]. Considerable work has been undertaken in New Zealand to address this problem, particularly regarding the use of video consultations [11-13].

### Rural Underserved Populations

The term *underserved* (also known as underresourced) population addresses situations where health care inequity exists because of system failures in health care delivery [14]. A major advantage of telehealth is increased access to health care for underserved populations, such as rural communities [15,16]; however, telehealth use during the COVID-19 pandemic was significantly lower in rural than in urban areas [17]. Rural areas are typified by low population density and less infrastructure than urban areas, with greater distances between services and people making it harder to deliver health services in rural areas [18]. In New Zealand, 1 in 4 people live in rural and semirural areas, with children, older adults, and Māori (the indigenous people of New Zealand) forming the greater proportion [19]. Owing to rural downturns and urban migration, the proportion of older adults in rural populations has been increasing at a faster rate than in urban areas [18]. The most deprived areas of New Zealand are rural [20], and ensuring equity in health care for rural New Zealanders is a priority [19,21].

### Older Adults

Similar to many high-income countries, the population of New Zealand is aging, and the proportion of older adults aged ≥65 years is expected to increase to 22% by 2031, accounting for approximately 50% of government health expenditure [21]. Older adults have higher rates of long-term comorbidities and disabilities [21] and can benefit from telehealth [10].

Thus, the overarching research question for this project was *how can telehealth systems be designed and implemented in rural underserved populations to improve access to health care services in New Zealand*.

This paper presents the findings of this qualitative and exploratory project. This work adds to the academic body of knowledge by examining key barriers to and enablers of telehealth adoption within an underserved population—older adults in rural communities.

## Methods

### Overview

The project adopted a sociotechnical systems perspective for the development of health care technologies [22], recognizing that the adoption of new technologies involves an interaction between complex infrastructures and human behavior. A qualitative approach was used to determine the barriers to and enablers of telehealth in rural communities. Qualitative research techniques use interactive methods [23] and an approach that assumes that individuals see their reality from a set of values, attitudes, and beliefs that reflect their life experiences [24]. Group methods are an effective means of gaining such insights [25].

### Ethics Approval

The New Zealand Health and Disability Ethics Committee operating procedures did not require a Health and Disability Ethics Committee review for this project. Following Massey University Human Ethics Committee processes, this project was evaluated by peer review, including suitability from a Māori research perspective, and judged to be low risk. Therefore, a low-risk notification was made to, and recorded by, the Massey University Human Ethics Committee as per the university’s process [26]. Participation in the project was voluntary, and informed consent was obtained from all participants.

### Eligibility

Criteria for selecting the rural areas in which to hold focus groups were identified using a modified Delphi process with the authors and the project advisory group (Textbox 1). Four geographically rural regions that met these criteria were identified: regions 1 to 3, aligning with the top, middle, and lower areas of the North Island, and region 4, aligning with the top of the South Island of New Zealand. In addition, the selected areas aligned with 1 district health board per region; for a map of New Zealand health regions and district health boards, refer to the New Zealand Health Partnerships website [27]. New Zealand underwent significant health reforms from July 1, 2022, although the regions and districts remained the same [28].

Eligibility criteria for the participants are listed in Textbox 2. The participants were recruited through purposive convenience sampling, which aims to gather a range of perspectives from diverse rural communities. The age for participation was ≥55 years, rather than ≥65 years, to allow for the fact that Māori tend to experience higher morbidity and mortality at a young age than non-Māori [21].

**Criteria for region selection.**

**Rural community criteria**

- Geographic spread (three-quarters of New Zealanders live on the North Island)
- Collaborative rural community
- Existing researcher networks in the community
- Travel >30 minutes to the nearest permanent primary health care center

**Criteria for participant selection eligibility.**

**Participant criteria**

- Normally residing in the selected rural communities
- Aged ≥55 years
- Willing to participate and able to consent
- Could have used or had not used telehealth before

### Significance of Māori (Indigenous People) in the Research

In New Zealand, 1 in 4 people live in rural and semirural areas; children, older adults, and Māori contribute to the greater proportion of those who live rurally [19]. Māori are the indigenous population of New Zealand, accounting for 16.7% of the total population [29], with a higher proportion living in underserved areas and experiencing poorer health outcomes [19].

The New Zealand health and disability system, which includes health research [26], has obligations within its relationship with Māori under *Te Tiriti o Waitangi* (The Treaty of Waitangi). These obligations are contained in 3 principles—partnership, participation, and protection. Equitable access to health care and Māori self-determination with health and disability services form part of these principles; hence, health services must work together with Māori in the governance, design, delivery, and monitoring of health and disability services. Māori must be co-designers of the health system for Māori [25,30].

Purposive sampling was undertaken to ensure representation of different types of population groups within rural areas and, in particular, to ensure that the Māori voice was heard as per the Treaty of Waitangi's obligations. In addition to having Māori present in the community focus groups, 1 focus group was conducted on a *marae* (Māori meeting place), at the invitation of the local *iwi* (tribe), who had connections with participants from other Māori in region 1 (Northern Region). The success of this sampling strategy is reflected in the number of Māori respondents (29/98, 29.5% of the research population) exceeding the proportion of Māori in the general New Zealand population (16.7%) [29].

### Recruitment

Participants could bring a support person (of any age), and a translator was available if required. Individuals wishing to take part in the study but unable to attend the focus group in their region had the option of an individual interview via Zoom videoconferencing or phone. Focus group participation was voluntary, and informed consent was obtained from all participants. A thank you gift in the form of chocolates, petrol vouchers, or supermarket vouchers was offered. The focus groups were conducted by IH, CL, and SW in person (on location) from June to July 2021, and pivoted interviews were conducted by CL by phone during August to September 2021. The focus group discussions and interviews were transcribed in full.

The process of prior engagement provided an opportunity to discuss the purpose of the research and time for the participant to get to know the interviewer [31] and assist researchers in gathering thick, rich data for analysis [32]. Most participants who registered an interest in participating in the focus groups or phone interviews were contacted by phone or email by CL before receiving the participant information sheet, signing the consent form, and conducting the focus group or phone interview. Before the focus group was held on the *marae*, IH, CL, and SW were welcomed with a *pōwhiri* (a formal Māori welcome) before entering the *whare* (Māori meeting house), thereby providing the *tikanga* or the general foundation for the context of the work and observing the cultural norms of the Māori participants [33]. The importance of respectfully engaging with Māori in *their space* is integral to establishing a relationship of trust and acceptance of the researchers and for acknowledging the principles within the Treaty of Waitangi [33].

### Focus Groups

In total, 7 in-person focus groups were held (June to July) with older adults living in the 4 rural regions of New Zealand. The eighth focus group pivoted to interviews by phone (August to September) because of the COVID-19 national *lockdown*. Furthermore, 1 to 3 focus groups were held in 3 of the 4 rural regions, and the fourth region had interviews by phone. The focus groups were conducted at local community halls, with 1 being conducted on a *marae*, and lasted 1 to 1.5 hours.

Each focus group was split into 2—a table for those who had used telehealth and a table for those who had not used telehealth—and the tables were run simultaneously. The project funder required data on the number of participants who had and had not used telehealth within the regions. The research team ran the 2 tables separately to allow older adults who were less confident with technology, or had had less contact with the health system, to have a different level of facilitated discussion than those who were familiar with telehealth and were more comfortable using the technology. Participants could choose the table they were most comfortable joining, irrespective of telehealth use; however, whether the participant had prior telehealth use experience was noted within the information in the demographics questions in the survey attached to the consent form. Despite the separation into 2 groups (used or had not used), when the discussions on the separate tables were reviewed after the focus group, they were remarkedly similar, with no themes identified by 1 group only.

The focus group semistructured interview guide of 4 questions with prompts was loosely based on the modification of the Penchansky and Thomas [34] access dimensions by Saurman [35], with adaptation for telehealth. Respondents could deviate from the interview guide, provided the discussion or interview remained relevant to the research question of the study. The same set of questions was used for the focus groups and phone interviews. After each event, the researchers debriefed and iteratively reviewed the process of conducting the focus group, which was recorded using memos. IH and CL alternated between tables of those participants who had and had not used telehealth with each new focus group. SW joined the table with the most participants.

### Analysis

Both deductive and inductive approaches were used, with deductive coding drawing from the Saurman [35] modification of the access dimensions by Penchansky and Thomas [34]. Following Braun and Clarke [36], these concepts were used as an initial coding device to attract analysts’ attention to relevant aspects of the data and understand users’ ideas of telehealth use, not to test any framework.

The data set was analyzed as a whole, as well as according to those who had had a teleconsultation or not had a teleconsultation. An inductive analysis following the qualitative thematic analysis procedure of Strauss and Corbin [37] was then performed to identify feelings, attitudes, and perceptions and to understand the participants’ experiences of rural telehealth. A total of 2 members of the research team coded the study independently, and the authors coded responses with an intercoder agreement of 94% [38]. In the first pass—open coding—the authors holistically read each response. In the second pass—axial coding—the 2 authors jointly identified subthemes within the larger categories. Finally, in the third pass—selective coding—the authors searched the data for specific responses that illustrated the subthemes. Any issues concerning the identification of subthemes or the coding of an individual response were discussed and resolved by the 2 authors and by reference to the whole team.

## Results

### Overview

In total, 98 adults aged ≥55 years took part across 7 in-person focus groups from 3 geographical regions, with 1 focus group pivoting to 10 individual phone interviews. In addition, 3 phone interviews were held with participants who could not attend the focus group in their area but wished to participate in the project. Phone interviews via Zoom videoconferencing were attempted; however, the unstable connectivity did not allow for an uninterrupted interview with any of the participants where Zoom videoconferencing could be used.

### Demographics

General demographics are shown in Table 1, including those who had experienced telehealth consultations as per the selection criteria. The age range of the patients was 55 to 92 years. More women than men participated (68/98, 69%), similar to other web-based surveys, in which women tended to be more likely to self-select to participate [39]. No attempt was made to attract more participants who had used telehealth than those who had not used telehealth previously. The distribution of those who had used telehealth was almost 60% (58/98), and those who had not used telehealth comprised almost 40% (40/98) of the participant population. Māori accounted for 30% (29/98) of the participants.

**Table 1:** Participants by region and total.

Characteristics		Region 1: Northern Region^a^	Region 2: Midlands Region^b^	Region 3: Central Region^c^	Region 4: Southern Region^c^	Total
Age (years), range		55-82	56-82	55-82	60-92	55-92
**Gender, n (%)**						
	Men	10 (27)	2 (20)	6 (31)	12 (38)	30 (31)
	Women	27 (73)	8 (80)	12 (69)	21 (62)	68 (69)
**Ethnicity, n (%)**						
	New Zealand European	10 (27)	6 (60)	15 (89)	30 (94)	61 (64)
	Māori	25 (68)	4 (40)	0 (0)	0 (0)	29 (30)
	Other	2 (5)	0 (0)	2 (11)	2 (6)	6 (6)
Has used telehealth, n (%)		24 (65)	7 (70)	12 (67)	15 (45)	58 (59)
Not used telehealth, n (%)		13 (35)	3 (30)	6 (33)	18 (54)	40 (41)

^a^A total of 3 focus groups; 2 community focus groups and 1 focus group held on a *marae*.

^b^A total of 10 individual phone interviews (because of the COVID-19 pandemic national lockdown).

^c^A total of 2 community focus groups.

### Thematic Analysis

#### Overview

Data were entered into NVivo (version 1.6.1; QSR International). The grounded theory process was not used for this research; however, the grounded theory approach for the data analysis was used as it offered a systematic method of thematic data analysis, ideal for smaller data sets and generating rich descriptions and exhaustive coverage [40]. Comparative notes (memos) were used by the researchers as they conducted an iterative review after each of the focus groups and some of the phone interviews.

The overarching theme was enthusiasm and willingness to use telehealth from all communities who are keen to be involved in further research and implementation of telehealth systems, as evidenced by the following:

I’d love it if I could see him [health provider] by telehealth...and it would be much easier than having to drive for 3-4 hours

Region 4 participant

Each focus group included participants who had and had not used telehealth. The level of enthusiasm varied between those who had and had not used telehealth. It took time and discussion for some people to understand the potential benefits of telehealth for them. A few participants said that they would not use telehealth, either because of their overall health or disability or because they would always want to be seen in person. Very few participants who had experienced a telehealth consultation said that they were not keen to use it again; however, they all suggested areas of improvement. Therefore, participants fell into 3 groups: those who would not use telehealth, those who would probably use telehealth, and those who would use telehealth. The key message was that even those who had not tried telehealth would be willing to use it, although having had the experience of using telehealth resulted in a much more positive attitude toward telehealth and subsequent use. Therefore, providing opportunities to use telehealth in its broadest form would increase the success of telehealth systems in rural communities.

Two major themes emerged from a thematic analysis of the data, namely, trust and choice, each with further subthemes connected by equity (Textbox 3).

**Themes and subthemes connected to equity.**

**Trust**

- Technology and telehealth
- Ability to use telehealth
- Health care system

**Choice**

- Health service access
- Consultation type
- Telehealth deployment

#### Trust

Three subthemes were associated with participant discussions around trust: trust in the technology and telehealth system using that technology; trust in a person’s ability to use that technology; and, finally, trust in the health care system and its provision of care regardless of the mode of delivery.

##### Trust in the Technology and Telehealth System

This subtheme highlighted the need for end users to be able to trust that the technology used in telehealth systems would work when needed and with expected outcomes. A participant reinforced the need for consistency and reliability by saying the following:

The telehealth system has to work well otherwise people will start using it and you’ll lose them straight away [if it doesn’t work]

Region 4 participant

Connectivity was a major barrier or enabler for trusting telehealth. The issues reported with rural networks included a lack of connection to networks or unreliable and unstable networks that disconnect without warning. Participants expressed skepticism about dependable connectivity and the expectation that they would consistently be able to access health care via telehealth:

I don’t think we’re going to be able to do a lot of these things [telehealth] that we’ve just been talking about, until there is a vast improvement in the internet services,...If I leave this house, I can’t get any reception if I’m at the back of the farm, and once I leave this house, there’ll be no reception for about half an hour

Region 2 participant

Web-based banking and emailing were used in the discussions as examples of providing services on the web as participants were familiar with using them through rural network connectivity in their own areas. Alternative options to access web-based banking services (internet banking) were raised in each focus group and during many phone interviews. There was considerable uncertainty associated with managing financial transactions on the web. Connectivity was described as follows:

...it’s intermittent...I think I’ve sent that email and no,...I haven’t or, I thought I had paid that bill, and no I hadn’t paid it [because the connection dropped off]

Region 4 participant

Frustration was expressed because of issues specific to rural settings, such as the pending removal of copper wire (landline phone) in New Zealand leaving some people with no connection, frequent power outages that cut off cell phone towers, and preventing the recharging of mobile phones. The lack of a collective community approach to cell tower installation, focusing instead on individual and tourist connections, was a cause for concern in 1 region. The participants in 1 focus group explained this as follows:

...we had flooding for 4 days. Electricity went [power outage] straight away and that’s why a lot of people have got generators for freezers. During the flood, after 3 days the copper lines went down too, and there were no batteries in the tower to charge our phones

Region 3 participant

Most participants used mobile data or Wi-Fi by repeaters. Many had cheaper, older, basic mobile phones, PCs, tablets, or routers without the capacity for high-quality video calls or were on limited prepaid phone plans; therefore, the quality and speed of the video, audio, and text communications were poor. Moreover, a lack of planning by mobile and Wi-Fi networks when providing connectivity meant that network access was inconsistent, patchy, and completely absent, even on the same road. A typical example of issues that those living in rural areas experienced were expressed as follows:

...I’ve got 40 minutes to do my [online] banking on some days where it should take five [minutes],...and the presumption is that we can all be online to get all these good services, it is not realistic because of the [variable] connectivity

Region 4 participant

Access to rural connectivity in New Zealand is primarily through 2 formats: cellular technology through the use of mobile cell towers or base transceiver stations and wireless internet offered through a combination of wireless broadband linked (often by repeaters or network extenders) to a home-based Wi-Fi router as part of a local area network. Sometimes, the network backhaul (transmitting a signal from a remote site or network to another site) is supported by satellite and rural fiber broadband. The strength of the signal, either emitted by mobile cell towers or wireless broadband, is influenced by several factors. These factors include environmental conditions such as distance, weather, and obstacles; the technology applied, including antenna design, capacity, and frequency type; and the position where signal transmission towers or access points are placed. Owing to these factors, a person with a device capable of receiving signals from cellular or home-based Wi-Fi may experience significant fluctuations in signal strength when they move between rooms or locations on their rural property.

Similar international studies [41,42] have reported on broadband access challenges with telehealth programs for both rural and underserved populations. The shift to internet-based health consultations and associated increased reliance on internet connections because of the impact of the COVID-19 pandemic have further negatively affected telehealth use for those with existing health disparities.

The broadband initiative released by the Ministry of Business, Innovation, and Employment addresses some of these problems with both the Ultra-Fast Broadband Programme and the Rural Broadband Initiative. These 2 initiatives are currently on track to having 80% of New Zealanders with access to ultrafast broadband and improved rural coverage to 90% by 2025 [43].

Cost was another major issue, both to upgrade devices and access the network, with many participants, particularly from lower socioeconomic communities, reporting not being able to afford these costs and participants saying that their options were few:

It’s too expensive [upgrading to get better network access]

Region 1 participant

The final issue for trusting telehealth was security. The overall impression was that the participants trusted the system to be secure. However, a participant stated the following:

...You need to be wary of a lot of stuff going on the internet, such as scammers...[however] when you need expert medical advice,...don't worry about who's dealing with your security

Region 1 participant

Security features such as 2-factor authentication and maintaining updated software were a cause for frustration for some families as many rural families shared email addresses and devices, and updates and changes to the software itself were difficult. A gentleman related the following:

...I was fortunate because I registered for a patient portal first, and they won’t accept my wife on the same email address

Region 4 participant

##### Trust in a Person’s Ability to Use Telehealth

The second subtheme highlighted issues related to the ability to use telehealth systems by both consumers and providers. Age was not an indicator of digital capability, and participants reported varying comfort levels with different digital technologies across all age groups, not only for themselves but also for the need for health providers:

...to come up to speed [learn how to use technology]

Region 1 participant

That said, people with disabilities experienced greater difficulty in using telehealth than others. However, at the same time, technology was also credited with increasing access for some people living with disabilities. Texting was one such example:

[technology] has been an amazing thing for the deaf community

Region 3 participant

Participants reported a widespread lack of knowledge on both how to use digital technology to access health care and what digital technology is available to be used. For example, when discussing a patient portal, one of the participants was excited to learn that there were other options available to access health care:

So, I just need to contact the doctor and ask them about a portal, then I can see all my medical things on the report? This is just the best bloody thing that ever happened because I'm sick of ringing [them] back having just missed a call from the nurse

Region 1 participant

There were several ways in which participants addressed their lack of knowledge about using digital technology. Writing down instructions or using teaching videos were some ways in which they coped with their lack of trust in being able to use technology and telehealth:

I see these video clips a lot on YouTube. I have done quite a bit of learning online.

Region 3 participant

Others took help from younger family members or partners, which was not always ideal:

...When they finally come and visit, my children,...they take the phone off you, and do it for you...[which meant a lost opportunity to upskill with the use of a digital device]

Region 1 participant

Ongoing training was another suggestion. It was preferred to be provided collectively in the community rather than individually, although some participants had undertaken individual web-based courses. It was felt that having group training enabled people to support each other:

...And that's where community education kicks in..., bringing the people to a central hub and actually educating them,..., in a community, the buy-in comes from the community, the capacity comes from the community...

Region 1 participant

Finally, participants proposed having ongoing support available, for example, a support person in a local hub, community center, or health center or in a mobile van:

If the health centre had a little workshop, they [older adults] could do it along with a medical visit, where they can sit down with someone who can help [to learn about patient portals]

Region 4 participant

##### Trust in the Health Care System

The third subtheme highlighted concerns raised regarding trust in the health system. Some comments were positive, particularly the provision of emergency care (first responders and rescue helicopters), with participants supporting more funding for helicopters, in particular. However, others were negative, particularly regarding chronic care management and referrals between services. Waiting times and the lack of communication and coordination between providers such as general practitioners (family physicians and primary care providers) and community pharmacists were frustrating for many. One of the participants went further with their experience:

...To get the same GP you have to book up to one month ahead...and the last appointment I had with the GP, I got down there, and they hadn't let me know that she [the GP] wasn’t going to be there

Region 4 participant

Establishing and maintaining long-term relationships between consumers and providers were highlighted, with participants indicating that they, or members of their family, would rather travel a long distance to see their regular physician for consistent treatment rather than see a locum for the management of chronic conditions:

...They (family) want to have a face to face [in-person consultation]. They don't like having to see different people because you get given the wrong medication

Region 1 participant

Finally, the cost of accessing health care in general made telehealth a preferred option for some. One of the suggestions was as follows:

A number of health programmes could be provided online, and could be publicly funded...

Region 2 participant

This comment raised the need to discuss the funding of telehealth services, both at provider and consumer levels; telehealth may not necessarily be a cheaper option for the consumer once telehealth is established as a *business-as-usual* model of care. During the COVID-19 pandemic, and to date, the cost of telehealth services in New Zealand has varied throughout the country but has often been free to the consumer, giving a false impression of the true cost of accessing health care through telehealth.

#### Choice

Choice was the second major theme that emerged from the focus groups and interviews, with 3 further associated subthemes: health service access, consultation type, and telehealth deployment.

##### Choice in Health Service Access

This theme highlighted the considerations raised by participants in accessing health care services:

...Choice is important, cheques [banking checks] were discontinued, we had no choice

Region 3 participant

New Zealand banks stopped using checks after May 31, 2021, a contentious mandated decision that required a move to digital payment options, with which many older adults were unfamiliar.

Cultural appropriateness (safety) was a key idea that emerged, particularly for Māori. Participants indicated that health care services needed to be places where people felt culturally safe, supported, and with people with whom they have a good relationship:

They [patients] haven't even opened the door [to access the health service] because they feel uncomfortable. The result being that family members did not access the health services.

Region 1 participant

Therefore, some participants traveled more than an hour to see a preferred primary health care provider rather than one closer. One of the participants explained as follows:

...I just keep my GP, I drive two and a half hours each way [to see the same GP]...

Region 1 participant

Privacy was an important part of choice, irrespective of cultural norms and expectations. Having the option to choose who was present during a consultation was part of feeling safe with a health service, particularly for Māori, a collective-based society [25], who, in general, prefer having support people from their *whānau* (family) accompanying them to a consultation; however, some Māori participants indicated that they would prefer to be unaccompanied for privacy reasons. Hence, having the choice of a support person being present is important to meet Māori cultural needs and enhance their engagement with telehealth. However, some non-Māori participants indicated that they would also like to be able to choose whether and whom they could have present at a consultation, hence making this a choice option for anyone:

I don’t mind a [family] member there just helping and setting everything up [telehealth consultation]...[when talking with a doctor], but I really don’t need some of my whānau [family] knowing that I have a problem somewhere else.

Region 1 participant

Travel was another important determinant for accessing health care services. Some participants liked to combine a visit to health care services in the town with other activities such as shopping, visiting the library, performing social activities, picking up medications from the pharmacy, or having a blood test. The preferred mode of transport varied: some participants favored a rural bus service that went from their rural community to the local town, particularly if they were unable to drive, did not have a driving license, or were unable or unwilling to ask for a lift to town:

...bus options were also limited...it’s just the one bus [available bus service] but think of all the people who might potentially use the xxx bus, they’re mainly using friends and family. So, if you're [living] out here and you don't have a car, you would be having someone take you..., but how often can you ask your friends [to drive you]?

Region 4 participant

However, other participants did not like traveling on such a bus as it took up most of the day, and users found it very tiring. Some rural regions did not have a rural bus service, and without other options for transport, people stayed home and did not access any health care. One of the participants clearly stated the outcome:

...but I can’t make it [the travel], so I go without [healthcare]. A lot of us go without [health care]...they die!

Region 1 participant

Accessing specialist care was a major issue that was raised, and travel times could be up to 6 to 8 hours 1 way to see a specialist depending on road conditions, often requiring an overnight stay. The ability to have a specialist consultation via telehealth would mitigate the anxiety associated with driving long distances and navigating large cities. Telehealth was a popular option for accessing specialist services for almost all participants. One of the participants said the following:

If I’ve got to go [to see a specialist], I go the day before because I suffer really badly from anxiety. I can’t do long trips and I drive myself, so I’ve got to take someone [with me]...

Region 1 participant

Accessing mental health services was highlighted as another service in which telehealth could have a large impact:

Accessing mental health services...there is a level of stigma if you live in a small community—if you walk into a counsellor’s office, you feel exposed, so having the session from home means you have the comfort, and it may mean that treatment is sought, rather than not...and you can have loved ones with you sharing that time

Region 2 participant

Combining other services such as point-of-care testing, blood tests, and hearing and vision testing were suggestions for telehealth hubs, as well as medication delivery to home or to the site of telehealth services for collection. Banking services, where many are promoted to be accessed on the web or via regional branches with limited operating days and hours, and taxation services, where the main access is via email or automated phone answering services, are known to be problematic for older adults living in rural communities, particularly those with sensory loss or mobility issues. Options for a combination of mobile telehealth and non–health care services, with an accompanying technical support person to assist older adults with technical aspects coming out to rural communities, were well received.

An older adult participant shared with us why they would support mobile services:

I’m not game [confident] to do online banking, because all these years we’ve had a cheque book, [checking account] well, they’re gone now. I haven’t gone on online, but I’ve gone on to phone banking which, I don’t know how much safer it is

Region 2 participant

##### Choice in Consultation Type

The second subtheme was having a choice on how to have a consultation, for example, by video, phone, patient portals, text, or email. Having a choice of device —mobile phone, tablet, PC, and laptop—was also important:

I had a phone consultation with our pharmacist, it was absolutely fabulous

Region 3 participant

In fact, some participants were annoyed when they realized what telehealth was and that they had not been offered this as an option by their health care provider:

Out of the four consultations I've had since last March, one has been a phone [consultation], and the other three I’ve gone in [in person to see the GP], and I didn’t need to go in for any of those four [consultations]

Region 1 participant

Using telehealth for regular reviews was raised by many participants:

I would be happy to check in to the medical centre via video to make the check-ins 6 monthly rather than 3 monthly

Region 4 participant

Using telehealth to see whether an in-person visit was needed was also raised:

...if you had video, you could talk with the GP, you could show your husband’s swollen legs, or that he couldn’t move his arms,...or that doctor could look at his legs and say “take more frusemide” because he knows what medications he is on

Region 3 participant

Furthermore, using telehealth to provide access to services otherwise unavailable or inaccessible was raised:

I think it’s unreasonable for us to expect to have services on tap [instant access]. I think it all gets back to connectivity, and if I can talk to the physio on the phone or zoom and she can see how I'm going in my own house, I think that’s what we need

Region 4 participant

##### Choice in Telehealth Deployment

The final subtheme considered the deployment of telehealth services. For participants who were very comfortable using digital technology, there was a desire to use it from home. One of the participants said the following:

All the way through my [treatment], I have gone to only a couple of appointments, I never went to my GP here, ever,...I don’t want to be driving. Something clicked when this happened, and I thought I’m not going to use my energy for all of that [travelling]...I did it all on my phone, I wasn’t concerned about seeing their visual

Region 4 participant

However, most indicated that they would prefer some sort of *hub*, with the required secure technology and a technology support person available. Some communities indicated a preference for a fixed telehealth hub located at a community hall, primary care provider building, or local rural hospital. The repurposing of existing facilities was supported:

We have WiFi here at the meeting hall, we have set it up and that was one of the reasons to push on our side, was so that if we needed to have the doctor on [present on a telehealth call], he could link up [with us]

Region 1 participant

However, others suggested a mobile option for a telehealth hub, with a bus that travels around a set schedule of rural communities with a technology support person and maybe a nurse or health care assistant:

...my grandson had some teeth work done in the dental caravan, she [the dental nurse in the caravan] was able to be do a zoom conference to the dentist in the hospital

Region 1 participant

The idea of mobile health services is not new to New Zealand; there is a mammogram bus, surgery bus, and dental bus that travel around the country to different rural areas, and additional services such as a mobile echocardiogram service in areas with high rates of heart disease are planned. Thus, the idea of mobile telehealth services, either separate from or incorporated into existing mobile services is not unreasonable. The idea of staffing the bus with someone who could assist in the primary purpose of the bus in addition to providing digital technology support for the consumer was popular. For example, the mobile service could enable a telehealth consultation with a specialist located in another region and support the consumer through experience with digital education so that they may choose to undertake telehealth consultations in the future.

### Barriers and Enablers

Mapping these themes and subthemes to barriers and enablers is shown in Textbox 4. Often, an enabler is the opposite of a barrier; for example, one of the enablers is reliable connectivity—the opposite of this barrier is unreliable connectivity.

**Barriers and enablers.**

**Themes and subthemes mapped to barriers and enablers**

- Barriers
  Unreliable connectivity
  Cost—network, devices, and data
  Lack of access to devices
  Security or privacy concerns
  Low technology comfort level
  Low digital literacy
  Health service waiting times
  Poor communication
  Poor service coordination
  Lack of services
  Cost of health services
- Enablers
  Reliable connectivity
  Trust
  Having choice
  Flexible to individual or community needs
  Easy to use
  Support
  Training
  Reduction in travel
  Culturally safe
  Variety of deployment
  Access to a wide range of services (health and nonhealth)

## Discussion

### Principal Findings

The work reported in this paper is part of an exploratory project that investigates how underserved rural communities would like to use telehealth to improve their access to health services. In total, 7 focus groups and 10 interviews (pivoted from focus groups because of the COVID-19 lockdown) comprising 98 adults aged ≥55 years from 4 rural areas discussed their future needs for health services, how telehealth could improve access to these health services, and the barriers and enablers to using telehealth for their rural communities. Diversity was evident within the chosen rural communities, and all participants faced multiple challenges related to their access to health care services.

Rural communities are keen to adopt telehealth; therefore, the time is right to deliver health services by telehealth, although it needs to be implemented correctly the first time.

Although telehealth has been used in the health sector for decades, albeit in a limited capacity, the term *telehealth* is not well understood by consumers, and there is a lack of consumer awareness of the availability, benefits, and device options for telehealth. However, those who have used telehealth to access health services find it extremely helpful and would willingly use it again. Thus, providing opportunities for consumers to see telehealth in action and use it with appropriate support and digital literacy training would increase their understanding and awareness of telehealth and subsequently increase telehealth adoption.

The findings from this study align with the concept of co-design, identifying benefits for the user as opposed to the provider organization, leading to increased alignment with user requirements and user acceptance [44]. Partnership, participation, and active protection, the 3 principles within the Treaty of Waitangi [31], which includes the Māori voice in the design of telehealth systems *with rural communities* rather than *for rural communities*, can increase the successful use of telehealth to improve access to health care for different communities. The inclusion of the rural consumer voice will go somewhat toward addressing some of the inequity that exists with access to digital options for underserved communities.

The best choice for the mode of telehealth deployment—mobile digital technology bus, fixed venue, hybrid approach, or extending rural hospital capability—will vary among different rural communities, and there may be further solutions that have not been identified in this research. Combining telehealth services (either via mobile bus or fixed) at community venues with point-of-care testing or blood tests, hearing and vision testing, and social and health-based activities (such as mum and baby sessions, coffee or tea, wellness checks, and vaccinations), as well as allowing the use of telehealth technology to deliver access to nonhealth services such as web-based banking, would add value to telehealth systems and thus increase telehealth acceptance and use.

### Barriers and Enablers: Trust and Choice

The barriers and enablers shown in Textbox 4 align with other studies [4,45,46] and with the findings of previous studies by 2 of the authors who explored using sensor technology to support aging in place [47,48].

However, the themes of trust and choice have not been identified as enablers of telehealth. These themes, and their associated subthemes, align with the access dimensions discussed by Penchansky and Thomas [34], particularly those relating to connectivity and device availability, affordability, and accessibility. The addition of awareness by Saurman [35] is also clearly shown in this study, as many participants were unaware of both available health services and telehealth and of how to use digital technology that forms the basis of telehealth.

Trust is central to health care [49], easy to lose, and very hard to regain [50]. Implementing telehealth in an ad hoc manner that is difficult for users to connect to, with poor experiences, will not engender the needed sense of trust in the telehealth system. Hence, it is critical that telehealth implementations are culturally appropriate, well planned, sufficiently resourced, and have end user support. An example is the unreliable or absent connectivity experienced by the participants. The selected locations had some connectivity [51]; however, the participants’ experience was poor. This raises the issue that it is not just the existence of broadband coverage but also the quality of that coverage that must be considered to determine whether it is sufficient to sustain a telehealth consultation.

Choice requires having the option to select between ≥2 possibilities and, thus, that >1 possibility exists and is known. Individuals and rural communities face similar but different challenges to urban dwellers, and they already have a limited choice with broadband provider options and data speeds; hence, having a choice over how to access health services will enable them to better engage with telehealth. For example, having a choice over devices that fit their income or that can connect to the available broadband, or over telehealth services that are delivered in a way that makes them feel safe and welcomed, will enable people in underserved rural communities to engage with telehealth.

Importantly, building trust and choice into telehealth system design would result in telehealth systems that are culturally appropriate for First Nations’ people and indigenous populations who already experience significant inequity in access to health care and health outcomes [30,52,53].

### Limitations

Beyond the limitations inherent to the nature of conducting focus groups [54], although rich data in consistent themes were obtained, care should be taken with reproducibility and transferability of the findings drawn from the study and beyond the study locations and these rural underserved communities. As the number of focus groups and phone interviews grew, discussions with participants became transferable, and fewer new themes were raised; however, data saturation and the end of data collection are contentious and much debated topics with different definitions [55]. It is possible that other researchers may interpret the data into different themes or that further analysis might identify additional or different codes, as the nature of qualitative research is that it is subjective and therefore influenced by researchers’ personal biases [56].

Two main aspects determined the end of data collection for this project; available resources and the collection of sufficient data to enable meaningful, albeit subjective, analysis and inferences to be drawn by the researchers. The participants were not drawn from a random sample of individuals; rather, they actively volunteered to be involved in the study. The research was constrained in terms of the time to complete the project, with the added complication of needing to cancel 2 scheduled focus groups because of the second national COVID-19 pandemic lockdown and reverting from these last 2 focus groups to scheduling 10 separate phone interviews in an entirely new region. It could be argued that reverting to 10 separate phone interviews provided ≥8 hours to the transcription content and, therefore, even more credibility in obtaining thick, rich data from participants [32]. The team also had a cap on funding, limiting further exploration.

Finally, the impact that the COVID-19 pandemic may have had on recruitment, mode of engagement, and consumer attitudes toward telehealth must be considered.

### Conclusions

The participants from the underserved rural communities were keen to use telehealth to access health services but wanted more information about, and support to use, telehealth systems. Rural communities want to be involved in designing telehealth services available in their communities. Maintaining trust and supporting choice in the use of telehealth to access health care are key enablers of telehealth acceptance.
